# How to Select 2D and 3D Roughness Parameters at Their Relevant Scales by the Analysis of Covariance

**DOI:** 10.3390/ma13071526

**Published:** 2020-03-26

**Authors:** Stephane Tchoundjeu, Maxence Bigerelle, Francois Robbe-Valloire, Tony Da Silva Botelho, Frederic Jarnias

**Affiliations:** 1 Vallourec, 92100 Boulogne-Billancourt, France; Stephane.tchoundjeu@vallourec.com; 2 Laboratory of Industrial and Human Automation control, Mechanical engineering and Computer Science, UMR 8201, Polytechnic University of Hauts-de-France, 59313 Valenciennes, France; 3 Laboratory Quartz EA 7393, Supméca, 93400 Saint-Ouen-sur-Seine, France; francois.robbe-valloire@supmeca.fr (F.R.-V.); tony.dasilva@supmeca.fr (T.D.S.B.); 4 TOTAL Marketing & Services, Centre de recherche de Solaize, 69360 Solaize, France; frederic.jarnias@total.com

**Keywords:** roughness, lubricant, wear, pitting

## Abstract

In this paper, a multi-scale methodology is proposed to model and characterize the effect of two lubricants on changes in surface morphology during a running-in test. The test concerns two steels samples, mounted on a twin-disc tribometer to test each of lubricants A and B for a period of 42 h. The changes are characterized by the standardized roughness parameters given in ISO 25178. A technique involving replication is used to monitor wear during the test. Using all these replication measurements, a multi-scale methodology is applied. These selected models highlighted the relevant parameters for quantifying wear during lifespan, and also showed that lubricant A was better able to preserve surface integrity during wear than lubricant B.

## 1. Introduction

Many tribology studies have used surface topography to analyze and quantify the behavior of a system (wear, friction) [1,2]. In numerous cases, the study of a tribological process involves the continuous (or progressive) monitoring of the morphological change of the surface over time [3] or during the continuous evolution of a mechanical request: for example, monitoring surface damage on a tribometer [4], surface wear [5,6,7,8], changes in the tribo-chemical process [9,10], plastic deformation [11] of roughness asperities through an increase in macroscopic contact pressure [4,12], etc. In other words, the monitoring of surface morphology constitutes the basic analysis of the tribological process. In our case, we will characterize surface changes on the basis of the number of cycles through the most relevant roughness parameters. However, the study of the temporal tribological process becomes truly interesting if it is studied under different conditions. Such conditions could be environmental (temperature [13,14], corrosion [15], biological environment [16,17]) process conditions (lubricant type [18], mechanical request [19], etc.) or material conditions [20,21,22].

The objective of a tribologist is to quantify the tribological response of the system taken under these diverse conditions in order to understand the elementary mechanisms [23] so as to propose innovative solutions, such as finding an anti-cover wears [24,25], choosing a good lubricant [26], or determining the most resistant materials [27]. In our case, the surface morphology will be modified during running-in, but the nature of the surface response is going to depend on test conditions (lubricant type). Those morphological modifications, occurring during the running-in, are significative of the wear behavior of the surface during lifespan but are difficult to highlight since they are of order of magnitude of the roughness itself. The main task is to define a statistical treatment protocol adapted to this type of problem. The questions to be answered are:

- What are the roughness parameters and in what spatial scale (cut-off length) must they be estimated to characterize the state of wear of the tribological system [28]?
- How, morphologically, can the effect of the lubricant be characterized during wear [29]?
- Did wear depend on surface anisotropy [30]?

The 3D surface topography has brought a finer characterization in tribology by introducing new parameters [31,32,33,34] and has shown the importance of roughness in contact mechanics [35,36,37]. New concepts based on segmentation [38] have shown the role of texture on tribological behavior [39,40]. More recently, multi-scale analysis has shown the characteristic scales encountered in tribology [41,42], on the phenomena of machining [43], wear [44], grinding [45], abrasion [46], superfinishing [47], catastrophic wear [48]. Finally, methods based on information theory [49] have been used to characterize abrasive processes [50].

To answer these three questions, we will use a particular statistical tool known as. the analysis of covariance (ANCOVA). It is a statistical method that uses a general linear model to test the effect on a continuous dependent variable of one or several categories of independent variables, independently of the effect of the other continuous quantitative factors, named covariates.

## 2. Materials and Methods

### 2.1. Tribometer Bench Test

Materials were tested in the lubricated rolling-sliding situation, corresponding to gear teeth contact. Such solicitations can be performed on a simple specimen, mounted on a twin-disc device.

#### 2.1.1. The Bench Test

**Figure 1:**
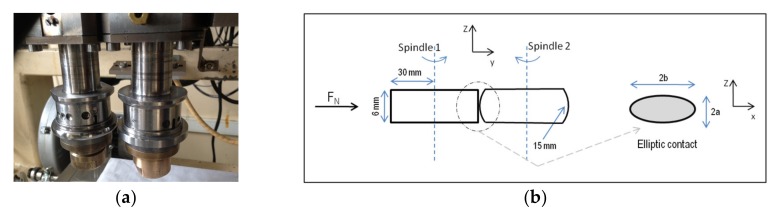
(**a**) The twin-disk tribometer and (**b**) the elliptic contact between both test specimens.

The test was carried out on a twin-disc tribometer (SUPMECA School, Saint-Ouen, France) [51,52]. Each disk is mounted on an independent spindle driven at a maximum speed of 10,500 rpm. A constant normal load is applied using a dead weight. A force sensor measures in real time the tangential force during the test. A thermo tank regulated at 40 °C—containing the lubricant—allows both tested discs to be completely immersed, and thus contact one another, during the test (Figure 1).

#### 2.1.2. The Lubricants

Two different oils, formulated from the same base, were used for the test:

- “Oil A”, known to not produce micro-pitting surface damage in rolling-sliding contact,
- “Oil B” known to produce micro-pitting surface damage in rolling-sliding contact.

#### 2.1.3. Test Disks

The tests were performed with two discs (one convex and one cylindrical) made of the same steel material (16MnCr5). Between these two discs, there is an elliptical Hertzian contact (Figure 1).

#### 2.1.4. Test Conditions

Table 1 groups all the conditions for the tests performed on the two-disc tribometer. From these conditions, we can define the conditions for running the bench test (Table 2). The ellipse radii as well as the local pressure during contact are determined by considering Hertzian contact behavior. The minimum lubricant film thickness is determined using Hamrock and Dowson’s equation [53].

**Figure 2:**
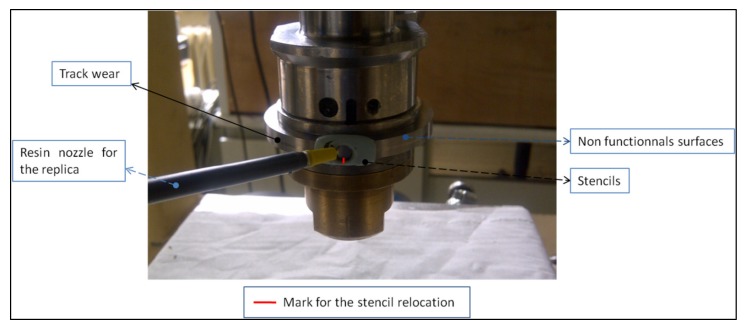
Replica method.

The test is interrupted at intervals (1 h, 2 h, 4 h, 6 h, 8 h, 12 h, 16 h, 20 h, 25 h, 30 h, 36 h, and 42 h). These interruptions make it possible to monitor changes in surface roughness as well as to preserve these surfaces at these various stages, owing to the replication methods. Every lubricant test is duplicated with an “opened” stencil (as shown in Figure 2) to verifying that it is very repeatable and reproducible. The Table 2 below summarizing all the working conditions measured and monitored during the running tests.

#### 2.1.5. Measurement Methodology

These replications, shown in Figure 2, are composed of a fast polymerizing bi-component resin of silicone rubber for a close resolution of 0.1 μm [54]. This resin (RepliSet from Struers) is adapted for measurement using an optical interferometric microscope (interferometry) (SUPMECA School, Saint-Ouen, France).

The main problem involved is measuring roughness at a precise region of the surface and monitoring its change over time, which involves performing this operation at the same area at all times. This is why it was essential to set up a methodology that took into account, on the one hand, the relocation of the surface to perform the replication in exactly the same location, and, on the other hand, the repositioning of the measurement under the interferometric microscope as well. By means of a hard resin (different from the replication resin), we created stencils to relocate the replication of the surface on the discs without dismounting the contact. These stencils fit the shape of the disc so as to facilitate its repositioning on the disk. The contact between the two discs generates a wear track, with a width close to 0.4 mm according to the elliptic contact dimensions. Considering a 3 mm diameter replicate, there is 2.6 mm of non-worn surface which will be used to make the positioning mark for the replication. The measurement area on the interferometric microscope measuring 0.9 × 1.3 mm is centered on the one hand, owing to the wear track, and on the other hand, owing to the engravings made on the non-worn regions (red mark on Figure 2). These procedures made it possible to replicate the same region on a surface, as well as to measure and monitor its changes over time with a positioning accuracy estimated at 0.1 mm.

### 2.2. Roughness Analysis

#### Roughness Measurement

**Figure 3:**
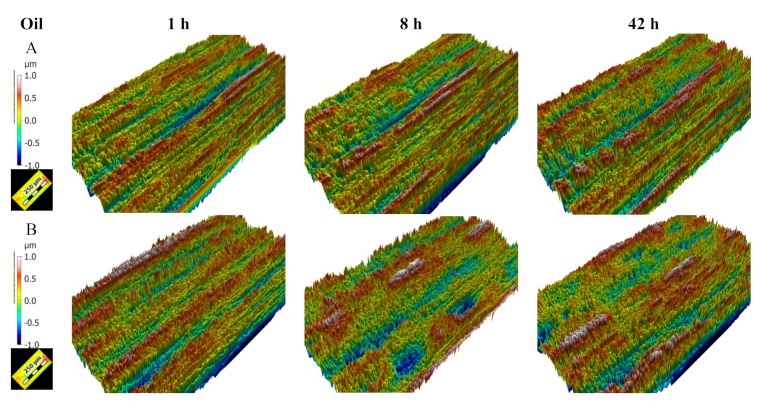
Evolution of surface topography after 1, 8 and 42 h of rolling-sliding test in lubricated conditions with respectively lubricant A and lubricant B.

Every replication is measured with the interferometric microscope and the surface topography can be observed using the MountainsMaps^TM^ software program (DigitalSurf, Besançon, France). Thus, we can compare topographical changes for both tested lubricants. The topography shown in Figure 3 is obtained by removing the shape and applying a high-pass filter with a cut-off length of 0.25 mm only to keep surface roughness. The topographical changes reveal that there is a tendency to significantly develop micropitting on surfaces tested with oil B (Figure 3). The next step was a multi-scale study aimed at finding the most relevant parameter to best characterize changes in micro-pitting during the test.

### 2.3. Relevance of the Best Roughness, Taking into Account the Effects of Both the Lubricant and Wear

#### 2.3.1. The Mathematical Concept

The main problem consists of finding what effect the lubricant will have on the apparition and evolution of micropitting. More precisely, what will the change in wear rate be when the tribological system is lubricated by lubricant A rather than lubricant B? This issue can be summed up by posing a mathematical relation between an appropriate roughness parameter (to be determined) and the number of wear cycles and statistically analyzed if the model coefficient(s) change with lubricants. Another analysis can be performed by analyzing the mean effect of the lubricant on the surface topography without taking into account the effect of wear rate. From a statistical perspective, covariance [55] will be used to measure the extent to which the wear rate coefficients change together and how strong the relationship is between them. To investigate this, an analysis of covariance [56], [57] will be performed. ANCOVA is a general linear model which blends ANOVA (analysis of variance) and regression [58].

#### 2.3.2. Mathematical Formulation

To understand how to use analysis of covariance in the field of tribology, a two-dimensional surface roughness parameter (2D parameter) analysis will be used and applied to the tribological system described in the preceding sections. In the case of our model, the effect on topography thus becomes an effect of the linear continuous variable (number of cycles) and a discrete variable (choice of oil). Note that the linear relationship in our case depends on the choice of oil. In other words:

Roughness = Constant + Oil Effect + a (Oil)* Number of Cycles

where a is the slope of the linear relation that depends on the oil.

From a mathematical point of view, analysis of covariance models is in fact simply a particular type of linear regression model in the following form and then the following mathematical model can be proposed for our tribological system:

(1) $$S_{k,\varepsilon ,\lambda }\left(w_{j}\left(o\right)\right)=\mu +\alpha_{O}+\beta \left({\bar{w}}_{j}-{\bar{w}}_{o}\right)+\gamma_{O_{1},O_{2}}\alpha_{O}\beta \left(w_{j}\left(o\right)-{\bar{w}}_{o}\right)$$

where:

$w_{j}\left(o\right)$ denotes the wear rate of the piece taken at time *j* and lubricated by Oil O (oil A or B).

${\bar{w}}_{o}$ denotes the mean wear rate corresponding to all experimental sampling times *j*.

$S_{k,\varepsilon ,\lambda }\left(w_{j}\left(o\right)\right)$ represents the value of *k*^th^ roughness parameter *S_k_*, evaluated at a given spatial scale ε (filter cut-off) with a λ filter (in our case, Gaussian high-pass and low-pass filters) on samples taken at time j and lubricated by Oil O (oil A or B).

μ represents the mean of $S_{k,\varepsilon ,\lambda }$ for all times and oils.

$\alpha_{O}\;$ quantifies the effect of oil O (oil A or B) on $S_{k,\varepsilon ,\lambda }$ for all wear times.

$\beta_{O}\;$ represents the slope of the linear relations between $S_{k,\varepsilon ,\lambda }$ and wear time with oil O (oil A or B).

$\gamma_{O_{1},O_{2}}\;$ represents the interaction between the two oils, A and B, and the mean wear rate.

#### 2.3.3. Different Cases Encountered in This Tribology Study

**Figure 4:**
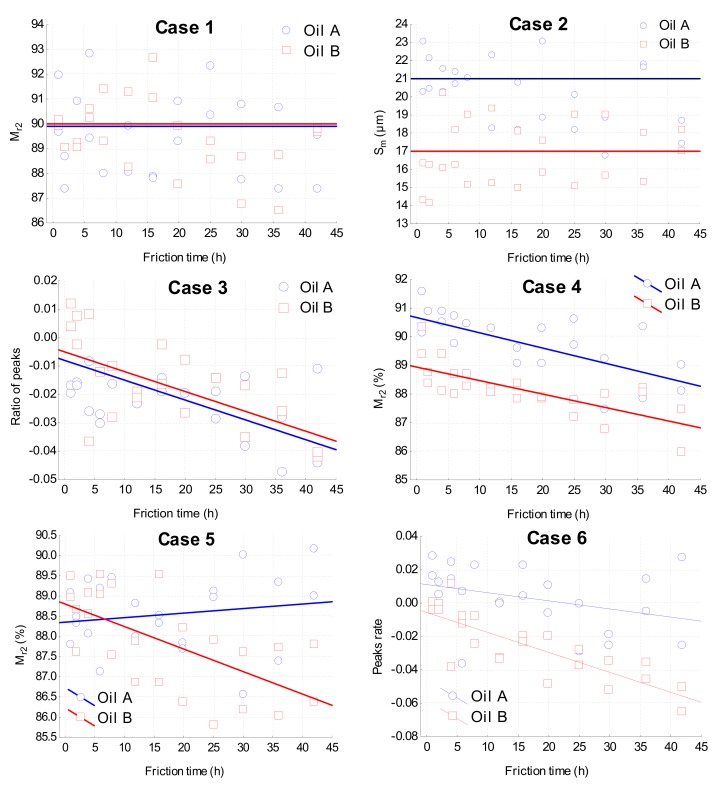
The different cases of ANCOVA analyses.

In this part, we analyze the experimental results obtained from our samples for all cases met in tribological experiments. Even though the values are taken from our experimental measurements, only the different typical cases will be described, without explaining the results. Hence the roughness scale and the filter used will not be discussed. In this case, 2D roughness parameters will be analyzed (Figure 4). It is pointed out that the roughness parameters according will be analyzed to the standard ISO25178-2 and where further details on the parameters can be found.

- *Case 1: parameter M_r2_*. (material ratio delimiting the valley area). Here is no relation between roughness parameters $S_{k,\varepsilon ,\lambda }$, regardless of wear time and lubricant oils. In this case, roughness parameter M_r2_ does not characterize both friction time effect and oil effect and cannot be used to characterize any of the parameters of our tribological system (at this scale of the specified filter). So $S_{k,\varepsilon ,\lambda }\left(w_{j}\left(o\right)\right)=\mu$, i.e., $\alpha_{O}=0,\beta =0,\gamma_{O_{1},O_{2}}=0$
- *Case 2: parameter S_m_.* (Distance between asperities) There is no wear effect, but only a lubricant effect. This case corresponds to physico-chemical effects that modify surface topography differently for oil A or oil B, independently of wear over time (no tribo-corrosion effect). Roughness parameter S_m_ characterizes no wear effect, but the oil effect. More precisely, the oil effect means the level of roughness characterized by parameter S_m_ because the distance between asperities will change, but this distance does not show variation during the wear process. So, $S_{k,\varepsilon ,\lambda }\left(w_{j}\left(o\right)\right)=\mu +\alpha_{O}\alpha_{O}\neq 0\;$ with $\alpha_{O}=\alpha_{A}\;$ for oil A and $\alpha_{O}=\alpha_{B}\;$ for oil B and $\beta =0,\gamma_{O_{1},O_{2}}=0$.
- *Case 3: parameter ratio of peaks*. (2(peaks-valley)/(peaks+valleys)) There is no oil effect; there is only a wear effect. The surface morphology characterized by the ratio of peaks allows us to define the wear effect, no matter what oils are used in the tribological system. So, $S_{k,\varepsilon ,\lambda }\left(w_{j}\left(o\right)\right)=\mu +\beta \left({\bar{w}}_{j}-{\bar{w}}_{o}\right)$
  , $\alpha_{O}=0$ with β≠0.
- *Case 4: parameter M_r2_(%)*. There are both oil and wear effects but the friction time effect does not depend on the oil. More precisely, the morphology characterized by parameters M_r2_ make it possible to describe the friction time effect but the linear tendency is not different for the two oils. However, the nature of the oil changes the morphology characterized by this parameter. It can be noticed that this 2D parameter M_r2_ is the same parameter as that used to illustrate case 2. However, this parameter is not evaluated on the same scale for both cases, meaning that the scale for evaluating the topography influences the choice of a tribological model and consequently a multi-scale roughness analysis is required. In this case, $S_{k,\varepsilon ,\lambda }\left(w_{j}\left(o\right)\right)=\mu +\alpha_{O}+\beta \left({\bar{w}}_{j}-{\bar{w}}_{o}\right)$ with β≠0, $\alpha_{O}\neq 0$ with $\alpha_{O}=\alpha_{A}$ for oil A and $\alpha_{O}=\alpha_{B}$ for oil B.
- *Case 5*. There is no oil effect; there is only a wear effect that depends on the oils. The interpretation of this statistical case in the field of tribology is a difficult task. This means that the oil does not affect the mean roughness but roughness changes during wear. However, as the friction time effect depends on time, mean roughness is also time-dependent and is therefore subject to measurement sampling rate. As a consequence, this case does not constitute a tribological relation; this is why it can be reduced to a borderline case for ANCOVA analysis. So, $S_{k,\varepsilon ,\lambda }\left(w_{j}\left(o\right)\right)=\mu +\beta \left({\bar{w}}_{j}-{\bar{w}}_{o}\right)+\gamma_{O_{1},O_{2}}\beta \left(w_{j}\left(o\right)-{\bar{w}}_{o}\right)$ with $\alpha_{O}=0,\beta \neq 0,\gamma_{O_{1},O_{2}}\neq 0$.
- *Case 6.* There are both oil and wear effects, but the friction time effect depends on the oil. This is the most accurate model in tribology. In our case, this model makes it possible to quantify the effect of a lubricant on the friction time effect. As a consequence, this model characterizes the different lubrication mechanisms involving a change in surface morphology and therefore guarantees surface integrity. So, $S_{k,\varepsilon ,\lambda }\left(w_{j}\left(o\right)\right)=\mu +\alpha_{O}+\beta \left({\bar{w}}_{j}-{\bar{w}}_{o}\right)+\gamma_{O_{1},O_{2}}\alpha_{O}\beta \left(w_{j}\left(o\right)-{\bar{w}}_{o}\right)$
  . $\alpha_{O}\neq 0$ with $\alpha_{O}=\alpha_{A}$ for oil A and $\alpha_{O}=\alpha_{B}$ for oil B, $\beta \neq 0,\gamma_{O_{1},O_{2}}\neq 0$.

### 2.4. Multi-Scale Roughness Characterization

**Figure 5:**
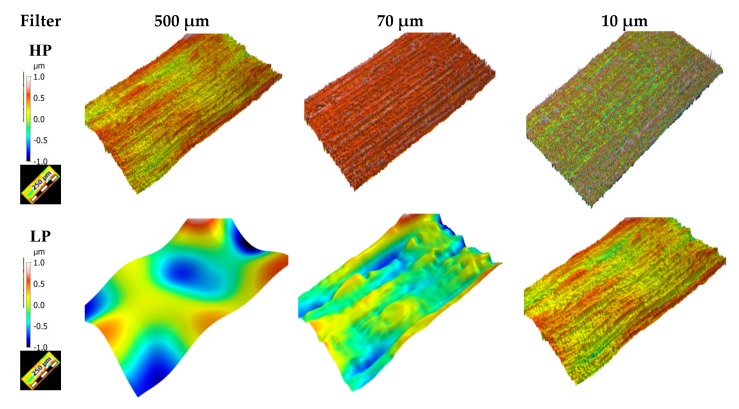
Multiscale decompositions of worn sample after 5 h using lubricant A with high pass filters and low pass filter with cut off or the surface decomposition with two cut-off corresponding to 10, 70, 500 µm. These cut-off are chosen arbitrary to visualize the multi-scale transformation of surfaces.

The Gaussian filter has been recommended by ISO11562-1996 and ASMEB46.1-1995 standards for determining the mean line in surface metrology. This filter was adapted in order to filter 3D surfaces with a given cut-off value [59]. In this study, only the high-pass filter (HP) will be presented (for the sake of simplicity, we have omitted the results for the band-pass filter because the best parameters were not relevant in this study). Our procedure is used to filter all surfaces with different cut-offs in order to obtain a multi-scale decomposition. Total of 30 consecutive steps are used in this decomposition, with a cut-off varying between 2 µm and 500 µm. Figure 5 represents thee multi-scale decompositions of a worn sample after 5 h using lubricant A with high-pass filters (HP) and low-pass filters (LP) with a cut-off or surface decomposition with two cut-offs corresponding to 10, 70, 500 µm (8).

When the cut-off length decreases, microscopic details appear on the filtered surfaces. Then 3D roughness parameters are computed. 3D roughness parameters [60,61,62] are defined by the following standards: ISO 25178 defines 30 parameters, EUR 15178N also defines 30 parameters, but some are identical to those of ISO 25178. Only 16 parameters are the latest ones, however Sz (maximum height of surface roughness) and Std (texture direction) are calculated differently in both standards. Further, 7 3D roughness parameters related to surface flatness are defined by ISO 12781 and ASME B46.1 defines 7 similar parameters as the ISO 25178 standard (with different predefined filters) and one new parameter SWt (area waviness height). This gives a total of 56 different 3D roughness parameters, which will be considered in this study [61,62]. The 3D roughness parameters (see Table 1) can be classified into the following groups: amplitude parameters, spatial parameters, hybrid parameters, functional parameters, feature parameters, other 3D parameters.

### 2.5. Relevance of the Parameters

To measure the relevance of the roughness parameters calculated at a given spatial scale, the above-mentioned ANCOVA is used. For each evaluation length, all of these influences are calculated by linear fitting. From them and for each parameter of the ANCOVA model $p=\left(\alpha_{O},\beta ,\gamma_{O_{1},O_{2}}\right)$ the between-group variability and within-group variability (corresponding to estimation errors for the roughness parameter of each group) are calculated [63]. For this case, we use the Fisher statistic noted F. The F value is used in statistics to practice F-test in different models such one-way analysis of variance. This test is used to assess whether the expected value of a quantitative variable within several pre-defined groups (ANOVA) and/or relations (ANCOVA) differ from each other avoiding multi-comparison. Briefly, the F represents the ratio of the explained variance/unexplained variance. The explained variance can be sum up as a measure of the discrepancy between a model and experimental data. In other words, it is the part of the model’s total variance that is explained by factors/relations that are supposed to be present and that is not due to error variance (the unexplained variance). Higher explained variance (and then F value) is, stronger strength of association is and predictions will be more accurate. Applied in our formalism, the result, denoted by $F\left(S_{k,\varepsilon ,\lambda },p_{i}\right)$, is the ratio produced by dividing the “between-group” variability by the “within-group” variability of roughness parameter $S_{k,\varepsilon ,\lambda }$ for parameter $p_{i}$ [64]. In other words, this result compares the effect of each process parameter on the roughness parameter value with its estimation error. Consequently, for a given process parameter, a value of $F\left(S_{k,\varepsilon ,\lambda },p_{i}\right)$ near 0 suggests the irrelevance of roughness parameter $S_{k,\varepsilon ,\lambda }$ estimated at evaluation length ε and filtering method λ to represent the effects of process parameter $p_{i}\;$ under consideration. The higher the value of $F\left(S_{k,\varepsilon ,\lambda },p_{i}\right)$, the more relevant parameter $S_{k,\varepsilon ,\lambda }$ is, estimated at scale ε. In this way, we can compare not only $F\left(S_{k,\varepsilon ,\lambda },p_{i}\right)$ with regard to the evaluation length but also to the chosen roughness parameter. By checking the highest value of $F\left(S_{k,\varepsilon ,\lambda },p_{i}\right)$, the most pertinent roughness parameter and its evaluation length can be selected to describe the influence of a given process parameter $p_{i}$ [65,66,67]. Two methods of analysis will be retained, the best global roughness parameter method (BGRP) and the best individual process parameter method (BGIP). The BGRP finds the roughness parameters that will best fit the Equation (2), the best F value for the total model.

In this method, the relevance of the total model is computed for a same scale k,ε,λ. Therefore, this analysis gives the single roughness parameter and its relevant scale that will best model wear and oil effects and their interaction. The BGIP finds the three best roughness parameters with their own relevant scales that possess the best $F\left(S_{k,\varepsilon ,\lambda },p_{i}\right)$ values.

## 3. Results

### 3.1. 3D Topography Analysis

#### 3.1.1. Best Global Roughness Parameter Method

**Figure 6:**
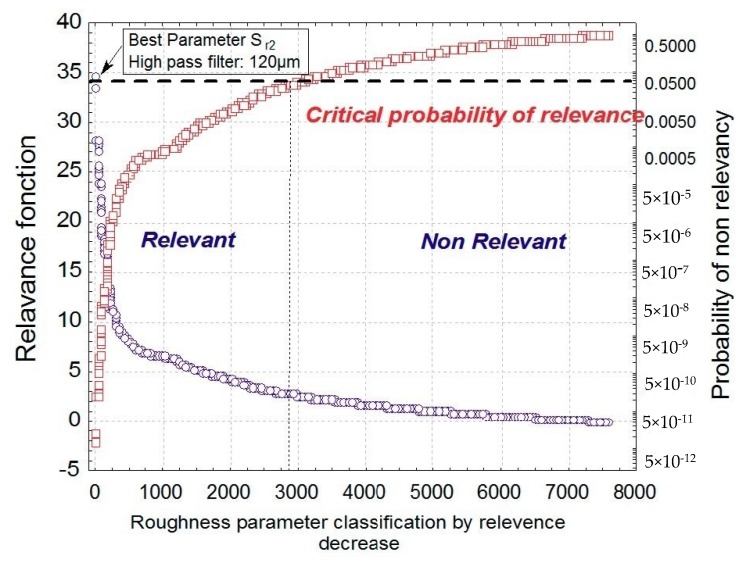
Plot of the relevance function F applied on the global model and their associated critical values (i.e., the probability of affirm fallacy that a relation exists although there is no relation) versus the position of relevance in descending order.

Let us now apply the global BGRP method to the tribological system. Figure 6 represents the changes in the Fisher variant F applied to our model as well as its critical value. We classify these values in decreasing F order and it can be observed that the best parameter is the first one; i.e., on the left side of the plot.

**Figure 7:**
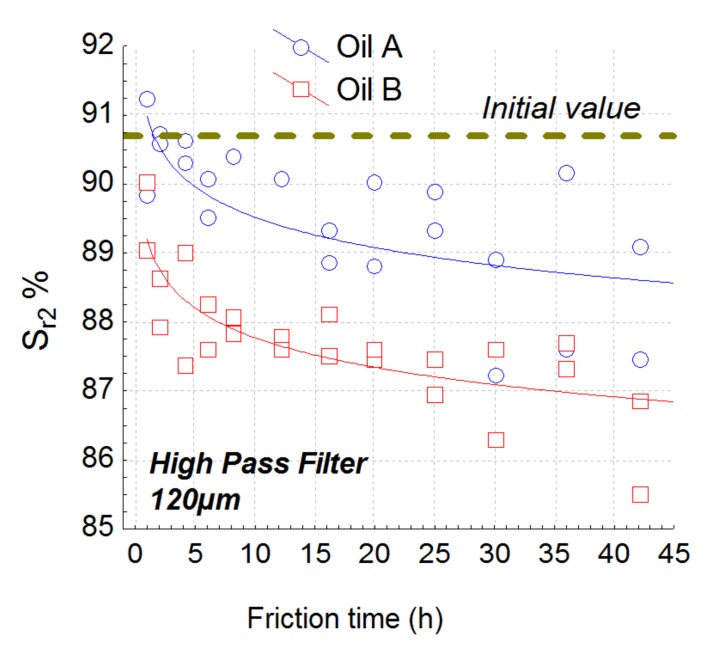
Plot of the most relevant parameters Sr_2_ obtained by the global methodology. This parameter is computed with a high pass filter with a cut off of 120 µm.

**Figure 8:**
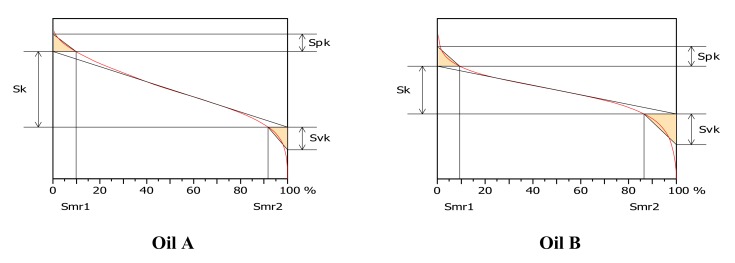
Abott–Firestone curve for a friction times of 36 h and high pass filter of 120 µm.

The best roughness parameter is parameter S_r2_ obtained with a high-pass filter with a cut-off of 120 µm (Figure 7) according to the model given by Equation (1). S_r2_ is a bearing ratio parameter representing the lower limit of the core roughness surface (Figure 8). After selection of relevance parameters and their associated scales with appropriate filters thanks to linear hypothesis of the ANCOVA model, the plot reveals that a nonlinear relation seems to be more appropriate to fit dependence of roughness parameter with wear rate allowing us to find asymptotical value of the roughness parameter for large wear rate.

**Figure 9:**
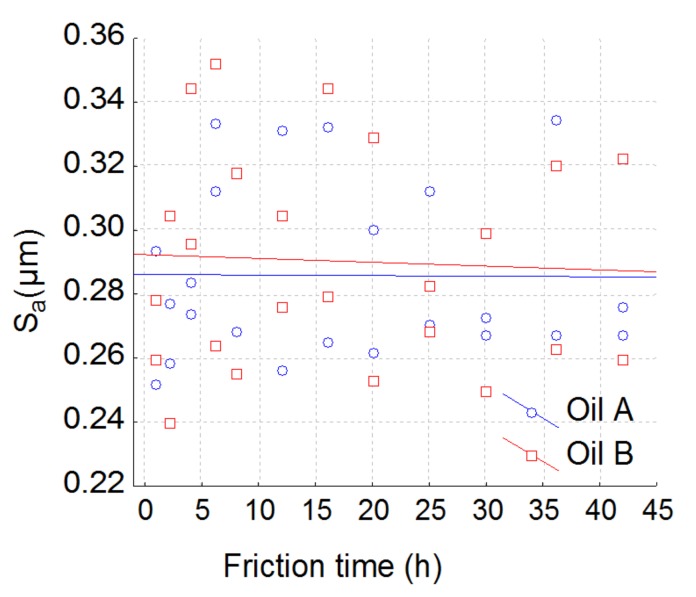
Plot of the most used parameters S_a_. This parameter is computed with a high pass filter with a cut off of 120 µm.

We notice that the depth of the valleys will increase during wear time. The Sr_2_ will present a significant decrease for oil B at the beginning of the wear process (pitting effect) and afterwards presents the same wear rate for both oils. To validate our methodology, S_a_ (the parameter most used in 3D topography) is represented in Figure 9. S_a_ represents the arithmetical mean height of the surface.

**Figure 10:**
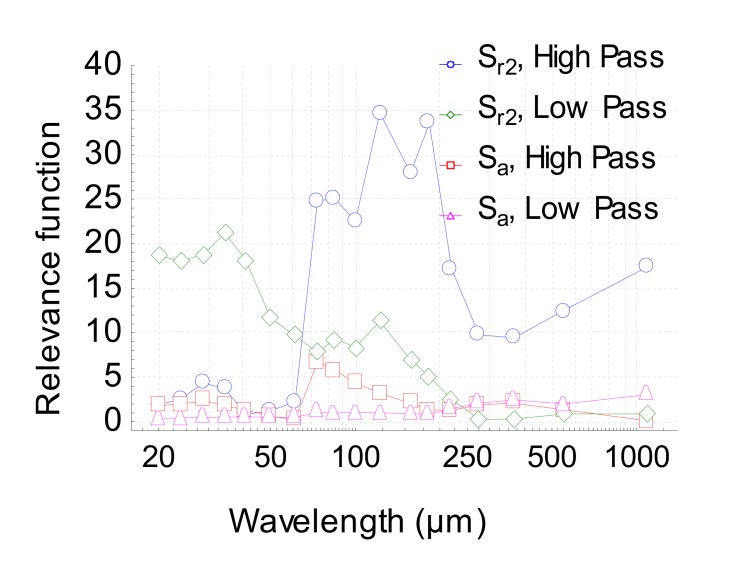
Plot of the relevance function $F\left(S_{k,\varepsilon ,\lambda },p_{i}\right)\;$ versus the wavelength for both parameters S_r2_ et S_a_.

S_a_ does not allow any discrimination according to the model given by Equation (1). One of the major goals of our method was to characterize the scale effects of the tribological mechanism. The aim of the proposed method was the ability to represent the scale effects observed. Indeed, the relevant function $F\left(S_{k,\varepsilon ,\lambda },p_{i}\right)\;$ can be plotted against the scale and makes it possible to quantify the range of the spatial scale on which the tribological process is relevant. Figure 10 shows that parameter S_r2_ is relevant on a scale range of 60 µm to 200 µm using a high pass according to the model given by Equation (1).

The topography of valleys under 60 µm is not affected and depends neither on the nature of the oil nor the wear intensity. On the higher scale (waviness), i.e., greater than 250 µm, the relevance function drops, meaning that the topography of deep valleys is not modified in the tribological system.

#### 3.1.2. Best Individual Roughness Parameter Method

**Figure 11:**
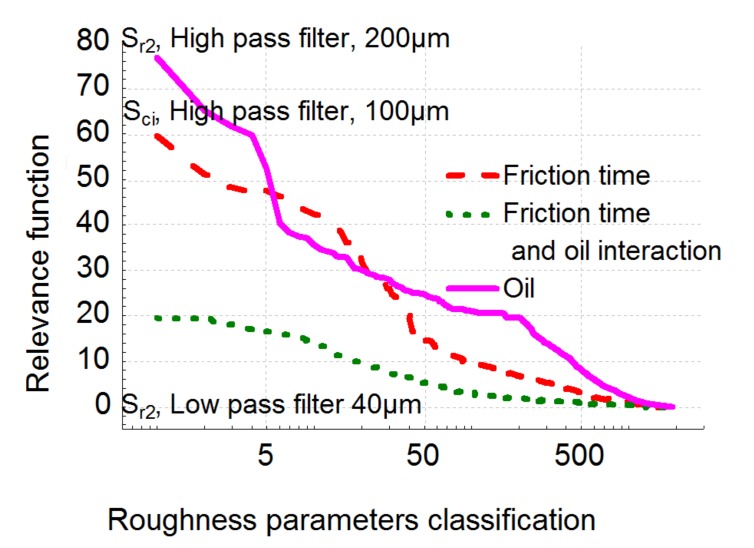
Plot of the relevance function $\;F\left(S_{k,\varepsilon ,\lambda },p_{i}\right)\;$ corresponding to oil effect, friction time effect and their associated interactions.

Let us now analyze the separate effects: wear and oil, and their mutual interactions. Figure 11 represents the relevant $F\left(S_{k,\varepsilon ,\lambda },p_{i}\right)\;$ function for both effects and their interactions. It can be observed that the maximum relevance value for the oil effect is given by S_r2_. This leads to the same conclusion as the global analysis given by the BGRP method (the scale range is quite similar in both analyses). Thus, the BIRP method makes it possible to quantify which process parameter (wear rate or nature of the lubricant) is the most relevant in the modification of the surface topography. In our cases, it can be shown that parameter S_r2_ quantifies the effect of oil in the process. Indeed, the plot shows that wear rate is quite similar but its level depends on the nature of the lubricant. Let us now analyze the wear rate effect on the surface topography.

**Figure 12:**
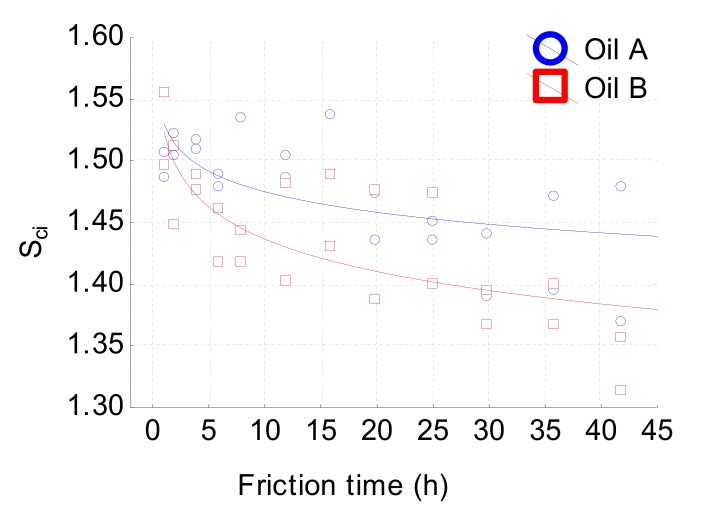
S_ci_ evolution versus friction time obtained by a high pass filter with a cutoff of 100 µm.

Figure 12 shows that S_ci_ effectively characterizes the wear rate according to the model given by Equation (1). This parameter represents the ratio of the void volume of the unit sampling area at the core zone (5–80% bearing area) over the RMS deviation. A larger S_ci_ indicates a good fluid retention, while a smaller S_ci_ denotes a smoother surface.

**Figure 13:**
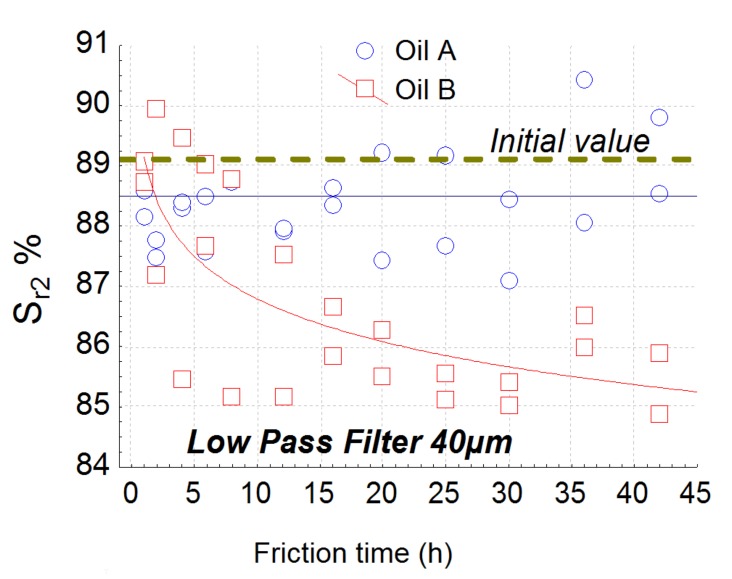
S_r2_ evolution versus friction time obtained by a low pass filter with a cutoff of 40 µm.

For a Gaussian surface, this index is about 1.56. During the wear process, valleys become deeper and deeper and the core of the roughness diminishes, which decreases lubrication efficiency. It can be noticed that the relevance scale of this parameter is of the same order as the S_r2_ one, confirming the strong correlation between deep valley formation and the drop in lubrication capability. Let us now analyze the interaction effect, i.e., how the oil effect changes the wear rate. This interaction is also given by roughness parameters S_r2_ for the low-pass filter with a cut-off of 40 µm. Figure 13 clearly shows the efficiency of the proposed methodology. Indeed, it can be observed that parameter Sr_2_ is slowly affected by lubricant B and highly affected by A during the wear rate measurement. This analysis emphasizes the fact that the same roughness parameters evaluated according to two different scales and different filter types (high or low pass) can characterize different tribological processes. Let us now analyze why the tribological system will be different with the two oils. Oil A does not involve a change in wear. By contrast, parameter S_r2_ will drastically diminish with oil B. Oil B will be more sensitive to valley depths on a low rough surface because by suppressing the high frequency of the roughness, this tendency does not appear and appears for oil A.

### 3.2. 2D Topography Analysis

Let us now analyze the relevance of the 2D parameters. In fact, in the 3D surface measurements, only parameter S_tr_ includes characterization of surface anisotropy. However, the surface shows anisotropy because of the grooves created by the tool machining process. Moreover, as the sliding direction is not stochastic, surface roughness will change during the wear process differently in the sliding direction (horizontal) or perpendicular to this direction (vertical measurement). For these reasons, horizontal and vertical profiles will be extracted from the 3D measurements. Then 2D roughness parameters will be computed and the same methodology as described previously will be applied [61,68].

#### 3.2.1. Best Global Roughness Parameter Method

**Figure 14:**
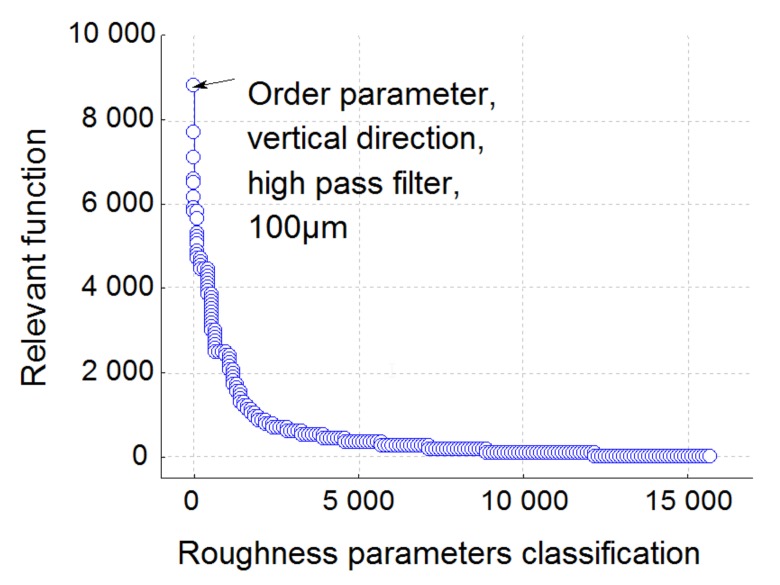
Relevance function for the Global model obtained from the 2D roughness parameters.

Order-parameter filtering by a high-pass filter is the most relevant parameter when evaluating in the direction of the sliding friction according to the model given by Equation (1) (Figure 14).

**Figure 15:**
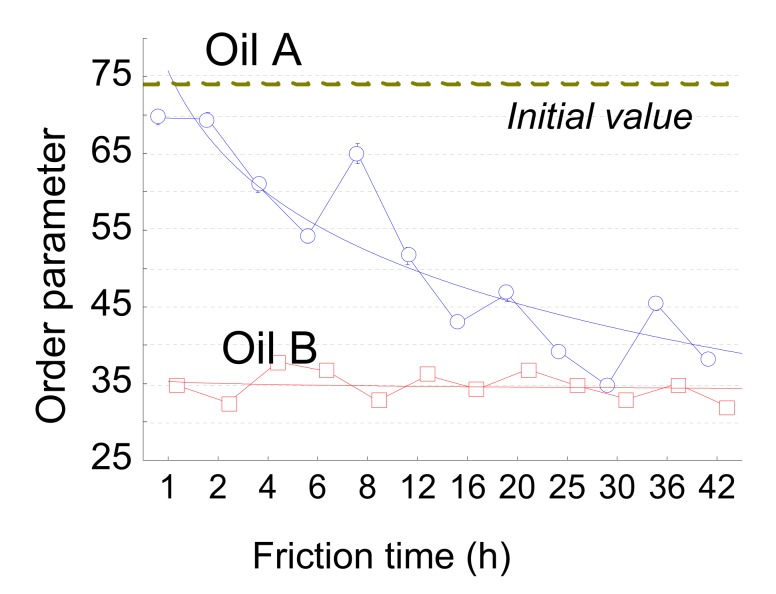
Order roughness parameters evolution versus friction time for the two oil A and oil B.

This parameter was proposed to quantify the regularity of a surface, independent of the amplitude and the scanning length units of the surface. This parameter lies between zero (perfect noise) and 100% (a perfect periodic surface). This parameter enables the identification of the anisotropy directions of regularity for a given surface. For a periodical surface, the greater the noise, the lower the anisotropy is. The regularity parameter can be used for several purposes: to identify the direction of periodical structures formed by laser-pulsed radiations on the surface of solid work pieces; to examine the reproducibility of surface machining methods such as finishing processes and to identify the surface regularity produced by abrasive machining, such as precision surface grain, abrasive slotting, and lapping. Figure 15 represents the order parameters (Appendix A) for the 2 oils, A and B. The order parameter decreases for oils A and B because of the depth attenuation of the initial grooves caused by the tool machining process. The order parameter decreases drastically for oil A and slowly for oil B. This parameter strongly discriminates the oil effect on the tribological system.

#### 3.2.2. Best Individual Roughness Parameter Method

**Figure 16:**
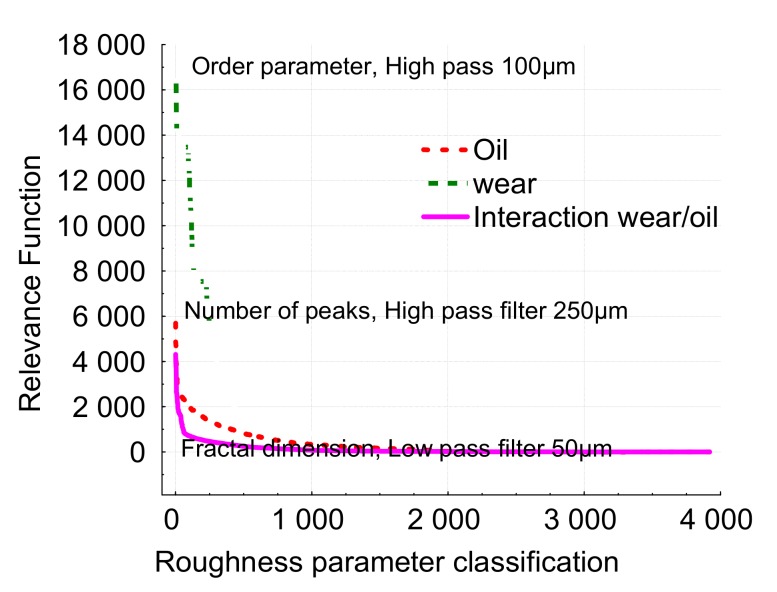
Plot of the relevance function $\;F\left(S_{k,\varepsilon ,\lambda },p_{i}\right)\;\;$ corresponding to oil effect, friction time effect and their associated interactions corresponding to the 2D roughness parameters.

The order parameter is the parameter that characterizes the wear effect (Figure 16). It is the same scale as the value given by the global analysis.

**Figure 17:**
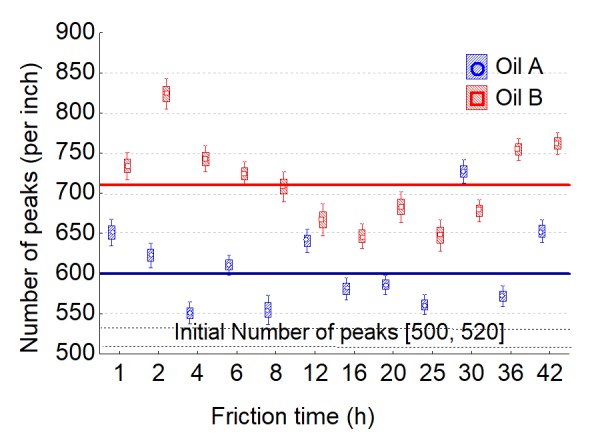
Number of peaks per inch evolution versus the friction time for both oils (A and B).

**Figure 18:**
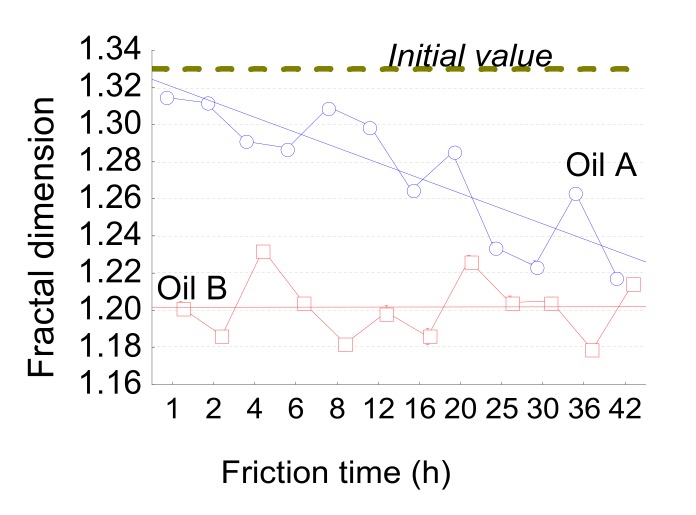
Fractal dimension evolution versus the friction time for both oils (A and B).

For the oil effect, the number of peaks in the horizontal direction is the most relevant parameter with a high-pass filter of 100 µm according to the model given by Equation (1) (Figure 17). In the beginning, oil B presents the higher number of peaks. This characterizes damage to the materials at a very small scale (high number of peaks around few µm). The initial surfaces get 500/520 peaks per inch. During the first instance of wear, peaks increase to 600 for oil A and 700 for oil B. These values seem to not depend on wear and can be linked to rapid initial damage. For the interaction, the most relevant parameters are given by the fractal dimension, but with a low-pass filter equal to 50 µm (Figure 18). This is characteristic of roughness irregularity on large range amplitude. The fractal dimension decreases for oil A and stays constant for oil B. At the end of wear, the fractal dimension of oil A reaches the same value as for oil B. The variation of this scaling law seems to show that scale effect is dependent on wear for oil A and not for oil B.

### 3.3. Topography Reconstruction at the Most Relevant Scale

This methodology of reconstruction is still under investigation. However, the results are of major interest for visualizing the topographical effects described by our analyses and will be helpful in the discussion of the results. The basic idea consists of recreating a surface from measured surfaces that represent the relevancy of the roughness parameter. To process on this analyses, the topographical maps are transformed into 32 bit grey levels images. One disposes of a set of morphological image treatments $O_{i}\left[\Theta_{i},w_{j}\left(o\right)\right]$ that can be applied to surface $w_{j}\left(o\right)\;$ given a new set of images (Equation (2)).

(2) $$\begin{array}{c} \Pi \left[\left(\Theta_{1},\Theta_{2},....,\Theta_{n}\right),w_{j}\left(o\right)\right]\\ \;=O_{n}\left[\Theta_{n},O_{n-1}\left[\Theta_{n-1},O_{n-2}\left[\Theta_{n-2},O_{\ldots }\left[\Theta_{\ldots },O_{2}\left[\Theta_{2},O_{1}\left[\Theta_{1},w_{j}\left(o\right)\right]\right]\right]\right]\right]\right] \end{array}$$

The new surface was then obtained by minimizing the function of Equation (3).

(3) $$\underset{\Theta_{1},\Theta_{2},....,\Theta_{n}}{\min}\underset{j}{\sum }F_{k,\varepsilon ,\lambda }{\left[S_{k,\varepsilon ,\lambda }\left(\Pi \left[\left(\Theta_{1},\Theta_{2},....,\Theta_{n}\right),w_{j}\left(o\right)\right]\right)-S_{k,\varepsilon ,\lambda }\left(w_{j}\left(o\right)\right)\right]}^{2}$$

**Figure 19:**
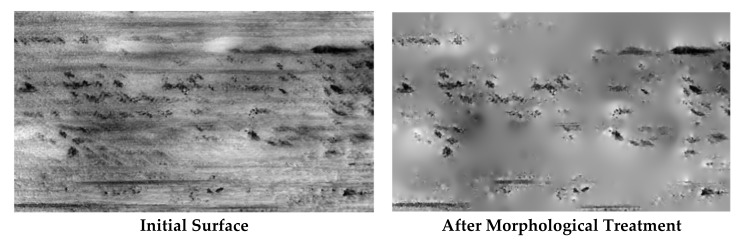
Morphological surface transformation according relevance of the functions.

**Figure 20:**
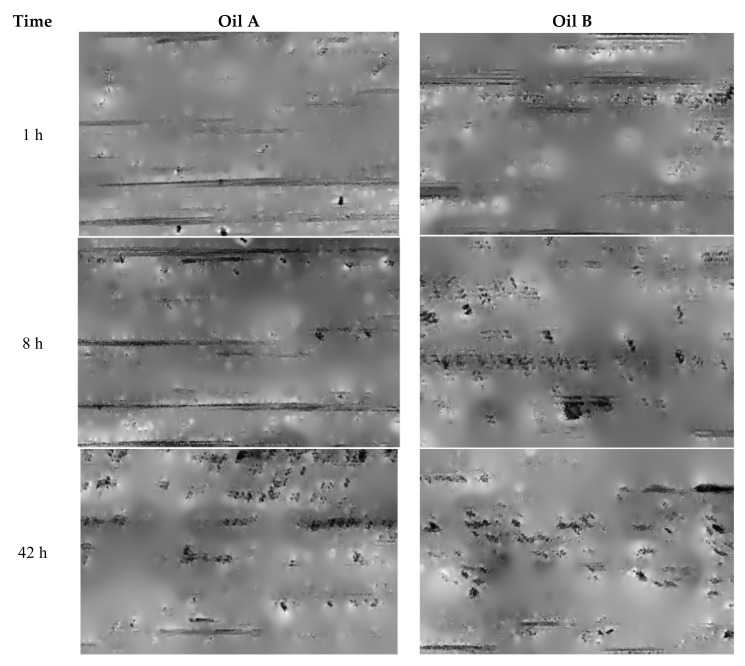
Evolution during wear of the reconstructed surfaces representing the relevance of the roughness parameters.

The idea is to simplify the surface such that the relevant parameters always remain the same. Figure 19 represents this transformation. As it can be observed, valleys clearly appear because of the fact that parameter S_r2_ is the most relevant parameter. It then becomes possible to visually analyze the changes in wear of both lubricants (Figure 20). As it can be observed, some pits appear around the size of 30–70 µm. But for lubricant A, pits appear progressively. At the ends of wear, the images seem to be quite similar, revealing that one has the same damage at the end of wear.

## 4. Discussion

During this study, we were able to use a surface replication technique to monitor, by interrupting the test, the topographic changes of surfaces during the running-in phase of a rolling-sliding twin-disc test in oil-lubricated conditions. We were able to determine the trends for the changes for every surface parameter under specified test conditions. The main advantage of replication is the possibility of analyzing a surface during a test, without proceeding to dismantle the samples on the bench. Unfortunately, the use of replication can be a source of a parameter standard deviation between the replicated surface and the real surface. This standard deviation has two explanations: first, dispersion caused by the use of the replication itself (hardening), and finally, by the exact relocation of the surface replicated. The analysis of the surface topography, at every interruption, thus imposes a relocation of the position to make the replication, so we can guarantee a rigorous comparison of the changes in the parameters during the test.

The study of parameter S_r2_ filtered with high-pass filter at 120 µm quantifies the load percentage of the surface at the border of the surface cavities. This parameter drops significantly for lubricant B in contrast to lubricant A. The changes in this parameter shows a decrease in load capacity, considering the increase of micro-scales on these surfaces. Concerning surfaces tested with lubricant A, the load capacity decreases more slowly, which results from the lower number of micro-scales on these surfaces. This tribological difference between the two lubricants occurs at around hour 6 of the test. From this moment, surfaces tested with lubricant B, present a significant loss of load capacity into the core part of the surfaces. This observation can be understood by the greater number of micro-scales on these surfaces, which leads to there being less material to guarantee the same percentage of surface load. In spite of the standard deviation for the values at the end of the test, it is possible to identify a specific behavior for each lubricant after the first 6 h of the test: time from which lubricant performances can be identified without waiting for the end of a long endurance test.

By analyzing the interaction between the oils and the wear effect, it was shown that S_r2_ filtered by a 40 µm low-pass filter is also the most relevant parameter (Figure 10). By analyzing the relevance function (Figure 9), the scale of relevance belongs to 20–40 µm and falls after 40 µm (relevance reaches the null value after 150 µm). At the 40 µm level, S_r2_ is quite constant at 88.5% for oil A and decreases for oil B until 85% (Figure 12). This interaction effect can be explained as follows: the decrease of S_r2_, observed with the high-pass filter decreases for both oils due to pitting (Figure 7). However, for oil A, pit diameters are less than 40 µm, no matter when the wear occurs. As a consequence, when passing over this critical length, oil A does not create pits wider than this size. On the contrary, oil B creates peaks up to a critical size of 100 µm. By suppressing forms with a low-pass filter, S_r2_ becomes irrelevant because pits less than 40 µm will disappear from the original topography.

This ANCOVA analysis is confirmed visually using the image reconstruction method (Figure 19). However, the common tribological process is given by the S_ci_ parameter observed with a 100 µm high-pass filter (Figure 10). The core fluid retention has same value for both oils and characterizes the lubrication dynamics. With friction time factored in, this index decreases from 1.5 to 1.35 (Figure 12). This clearly means that the fluid retention decreases with friction. In fact, as it can be observed on Figure 12, micropitting seems to locally reach a percolation threshold in terms of sliding friction and then reduces fluid retention.

2D roughness analysis makes it possible to more precisely investigate the anisotropy of the tribological effect. The order parameter estimated on a 100 µm scale with a high-pass filter perpendicular to the sliding direction is very characteristic of the tribological system. The order decreases progressively from 75% to 40 % with oil A and drastically drops with oil B to 40% (Figure 15). In fact, initial samples show grooves due to tool machining and are oriented in the direction of the rolling-sliding friction. The tool machining process will create some periodic topographical components perpendicular to the groove, i.e., perpendicular to the rolling-sliding friction. This is the reason that the initial order parameter value is very high (75%). During wear, the groove shapes will erode and the periodic components will decrease. For oil B, these groove shape erode rapidly. On the contrary, for oil A, theses groove shapes will erode more slowly. The lengths of these grooves are all less than 100 µm, which is equal to the most relevant filter cut-off. Groove erosion can clearly be observed on Figure 3.

The number of peaks clearly characterizes the oil effect. Pitting increases the number of peaks for both oils. This number is around 600 per inch for oil A and 700 for oil B (Figure 17). These parameters, given by ANCOVA to test the oil effect, are particularly relevant. Briefly summarizing, this roughness parameter characterizes the number of pits and not their sizes. As a result, the number of initial pits is given the first time that sliding friction occurs (germination) and is more pronounced (twice) for oil B than for oil A. This number will never change during wear; only pit morphologies change.

Concerning the fractal dimension, for oil A, the dimension linearly decreases with friction time and is quite constant for oil B. This parameter represents the multi-scale change of the surface morphology. This means that for oil B, the multi-scale morphological law remains constant. On the contrary, the multi-scale aspect will change progressively for oil A to reach the same value as oil B, at the end of the friction time. This can be explained by the fact that initial topography (which is highly fractal) will continuously disappear to reach low fractal topography for oil A and rapidly disappears for oil B to obtain the same morphological structure that characterizes the worn surfaces described by a low fractal dimension (1,2).

## 5. Conclusions

During this study, we developed and applied a new method for surface characterization through roughness parameters. Indeed, this method makes it possible to monitor wear in a tribological system without removing samples from the bench by the use of replication. Through this test on a twin-disc tribometer, we were able to highlight the advantages and the limitations of this replication method.

The ANCOVA-based method is a powerful tool for finding the relevant topographical scale, for dissociating the effect of a lubricant on the tribological system and, independently, the effect of friction time. ANCOVA was used first for determining the roughness parameter and spatial scale (cut-off length) that had to be estimated:

- To characterize the state of wear of the tribological system.
- The effect of the lubricant during friction time.
- The maximum change in wear rate during sliding friction due to the lubricant.

This methodology can be applied to many tribological studies, making it possible to quantify the effect of different environments on wear, friction, etc. It was shown that the use of both 3D and 2D roughness parameters made it possible to more precisely analyze wear effects by including the anisotropy effect (initial tooled surface, sliding direction, etc.,). The surface reconstruction method allowed us to visualize surface integrity by taking into account the relevance of the ANCOVA modeling.

## Figures and Tables

**Table 1:** Tribometer test conditions.

Test Conditions	
Normal force	600 N
Nominal rotation speed	6000 rpm
Temperature	40 °C
Sliding rate	30% the 30 first hours 40% the 12 last hours
Initial roughness	Sa = 0.4 µm

**Table 2:** Working conditions.

Working Conditions	
Thickness of the lubricant	0.95 µm
Hertz pressure	1700 MPa
Elliptic contact dimensions	a = b = 0.39 mm

## Appendix A. The Order Parameter

This parameter is due to Bigerelle et al. [69]. The main idea is to find a parameter without resorting to Fourier’s analysis because spectrum parameters have little robustness and sinus-cosinus basis is not always appropriate to characterize surface roughness. This parameter must have an upper limit value (100%) if surfaces are periodic, a medium value if surfaces get a non-neglected first-order autocorrelation and lower limit null value for uncorrelated random surfaces (white noise). First, we define a normalized autocorrelation function and find the integer *i* such as

(A1) $$R\left(i\right)=\frac{1}{Rq^{2}\left(N-i\right)}\sum_{j=1}^{N-i}y_{j}y_{j+i}>0.1$$

where y_i_ is equidistant discretized point in N points and *R_q_* is the well-known standard deviation of the amplitude. Second, we define an autocorrelation length L such as $L=x_{i+1}$ and a correlation integral J such as

(A2) $$J=\int_{x=0}^{x=L}R\left(x\right)dx$$

that represents a sort of fundamental as regards a function symbolizing a certain order power. Third, we define the K series *I_k_* of integrals

(A3) $$I_{k}=\int_{x=kL}^{x=(k+1)L}\left|R\left(x\right)\right|dx$$

that represents a kind of successive harmonics of the order power of profiles. Finally, the order parameter is defined as

(A4) $$order=100\sum_{i=1}^{K}I_{k}/\left(K\;J\right)$$

and lies between 0 (white noise profiles) and 100 (perfect periodic profiles without noise). The most important quality of this parameter is to be mathematically independent of the amplitude parameter and of the autocorrelation length of the surface. Then, the effect of the order of surface and the both scaled parameter amplitude and autocorrelation could be analyzed without correlation bias to study the scale effect.
